# A GDF5 Point Mutation Strikes Twice - Causing BDA1 and SYNS2

**DOI:** 10.1371/journal.pgen.1003846

**Published:** 2013-10-03

**Authors:** Elisa Degenkolbe, Jana König, Julia Zimmer, Maria Walther, Carsten Reißner, Joachim Nickel, Frank Plöger, Jelena Raspopovic, James Sharpe, Katarina Dathe, Jacqueline T. Hecht, Stefan Mundlos, Sandra C. Doelken, Petra Seemann

**Affiliations:** 1 Berlin-Brandenburg Center for Regenerative Therapies (BCRT), Charité – Universitätsmedizin Berlin, Berlin, Germany; 2 Berlin-Brandenburg School for Regenerative Therapies (BSRT), Charité – Universitätsmedizin Berlin, Berlin, Germany; 3 Institute of Anatomy, Dept. Anatomy and Molecular Neurobiology, Universitätsklinikum Münster, Münster, Germany; 4 Lehrstuhl für Physiologische Chemie II, Theodor-Boveri-Institut für Biowissenschaften (Biozentrum) der Universität Würzburg, Würzburg, Germany; 5 Department of Tissue Engineering and Regenerative Medicine, Universitätsklinikum Würzburg, Würzburg, Germany; 6 Biopharm GmbH, Heidelberg, Germany; 7 EMBL-CRG Systems Biology Program, Centre for Genomic Regulation, Barcelona, Spain; 8 Universitat Pompeu Fabra (UPF), Barcelona, Spain; 9 Institució Catalana de Recerca i Estudis Avançats (ICREA), Barcelona, Spain; 10 Institut für Medizinische Genetik und Humangenetik, Charité – Universitätsmedizin Berlin, Berlin, Germany; 11 Department of Pediatrics, University of Texas Medical School at Houston, Houston, Texas, United States of America; 12 Research Group Development and Disease, Max Planck Institute for Molecular Genetics, Berlin, Germany; Stanford University School of Medicine, United States of America

## Abstract

Growth and Differentiation Factor 5 (GDF5) is a secreted growth factor that belongs to the Bone Morphogenetic Protein (BMP) family and plays a pivotal role during limb development. GDF5 is a susceptibility gene for osteoarthritis (OA) and mutations in *GDF5* are associated with a wide variety of skeletal malformations ranging from complex syndromes such as acromesomelic chondrodysplasias to isolated forms of brachydactylies or multiple synostoses syndrome 2 (SYNS2). Here, we report on a family with an autosomal dominant inherited combination of SYNS2 and additional brachydactyly type A1 (BDA1) caused by a single point mutation in GDF5 (p.W414R). Functional studies, including chondrogenesis assays with primary mesenchymal cells, luciferase reporter gene assays and Surface Plasmon Resonance analysis, of the GDF5^W414R^ variant in comparison to other GDF5 mutations associated with isolated BDA1 (p.R399C) or SYNS2 (p.E491K) revealed a dual pathomechanism characterized by a gain- and loss-of-function at the same time. On the one hand insensitivity to the main GDF5 antagonist NOGGIN (NOG) leads to a GDF5 gain of function and subsequent SYNS2 phenotype. Whereas on the other hand, a reduced signaling activity, specifically via the BMP receptor type IA (BMPR1A), is likely responsible for the BDA1 phenotype. These results demonstrate that one mutation in the overlapping interface of antagonist and receptor binding site in GDF5 can lead to a GDF5 variant with pathophysiological relevance for both, BDA1 and SYNS2 development. Consequently, our study assembles another part of the molecular puzzle of how loss and gain of function mutations in GDF5 affect bone development in hands and feet resulting in specific types of brachydactyly and SYNS2. These novel insights into the biology of GDF5 might also provide further clues on the pathophysiology of OA.

## Author Summary

Mutations can be generally classified in loss- or gain-of-function mutations depending on their specific pathomechanism. Here we report on a GDF5 mutation, p.W414R, which is associated with brachydactyly type A1 (BDA1) and Multiple Synostoses Syndrome 2 (SYNS2). Interestingly, whereas shortening of phalangeal elements (brachydactyly) is thought to be caused by a loss of function, bony fusions of joints (synostoses) are due to a gain of function mechanism. Therefore, the question arises as to how p.W414R in GDF5 leads to this combination of phenotypes. In our functional studies, we included two reported GDF5 mutations, which are associated with isolated forms of SYNS2 (GDF5^E491K^) or BDA1 (GDF5^R399C^), respectively. We demonstrate that an impaired interaction between the extracellular antagonist NOGGIN (NOG) and GDF5 is likely to cause a joint fusion phenotype such as SYNS2. In contrast, GDF5 mutations associated with BDA1 rather exhibit an altered signaling activity through BMPR1A. Consequently, the GDF5^W414R^ mutation negatively affects both interactions in parallel, which causes the combined phenotype of SYNS2 and BDA1.

## Introduction

Growth and Differentiation Factor 5 (GDF5), which is also known as Cartilage-Derived Morphogenetic Protein 1 (CDMP1) belongs to the Transforming Growth Factor Beta superfamily (TGFB) and the subordinated group of Bone Morphogenetic Proteins (BMPs) [1]. GDF5 has a fundamental role during limb development, where it controls the size of the initial cartilaginous condensations as well as the process of joint development [2]–[4]. As a positive key regulator of early chondrogenesis, dimeric GDF5 initiates signaling by interacting preferably with two distinct BMP type I receptors, BMPR1A and BMPR1B, whereas binding via BMPR1B is favored over BMPR1A [5], [6]. Upon receptor phosphorylation, intracellular SMAD transducer proteins are activated in order to regulate target gene transcription [7], [8]. GDF5 activity is counteracted by BMP antagonists such as NOGGIN (NOG), which mask the receptor binding sites of GDF5 by a direct protein-protein interaction, thereby impeding receptor binding of the ligand and thus signaling [9], [10].

Alterations in GDF5 signaling due to specific point mutations have been associated with various diseases affecting bone and cartilage development [2], [11], [12]. Activating mutations in *GDF5* lead to a gain of function phenotype, resulting in increased chondrogenic activity as described for proximal symphalangism (SYM1, MIM #185800) and the multiple synostoses syndrome 2 (SYNS2; MIM #610017) [13]–[18]. The SYM1 phenotype is characterized by ankylosis of the proximal interphalangeal joints as well as fusion of carpal and tarsal bones. Additional symphalangism in the elbow and knee joint caused by *GDF5* mutations is a hallmark of the SYNS2 phenotype. In contrast to activating *GDF5* mutations, loss of function mutations result in hypoplastic or absent skeletal elements as described for the molecular disease family of brachydactylies. Depending on the affected phalanges, five different types of brachydactylies are categorized (A–E) including three subgroups (A1–A3) [11]. So far, mutations in *GDF5* have been linked to isolated traits of BDA1 (MIM #112500), BDA2 (MIM #112600) and BDC (MIM 113100) [16], [18]–[21]. Extreme shortening of digits and limbs are caused by homozygous loss-of-function mutations in GDF5, which are associated with different types of acromesomelic chondrodysplasia (Grebe MIM #200700, Hunter Thompson MIM #201250, Du Pan MIM#228900) [22].

Here we describe a family carrying a mutation in the mature domain of GDF5, p.W414R, showing combined clinical features of BDA1 and SYNS2. In this work we unravel the unique pathomechanism behind GDF5^W414R^ and thus demonstrate how one mutation in GDF5 confers a gain- and loss-of-function phenotype simultaneously.

## Results

### GDF5^W414R^ results in SYNS2 and BDA1

**Figure 1:**
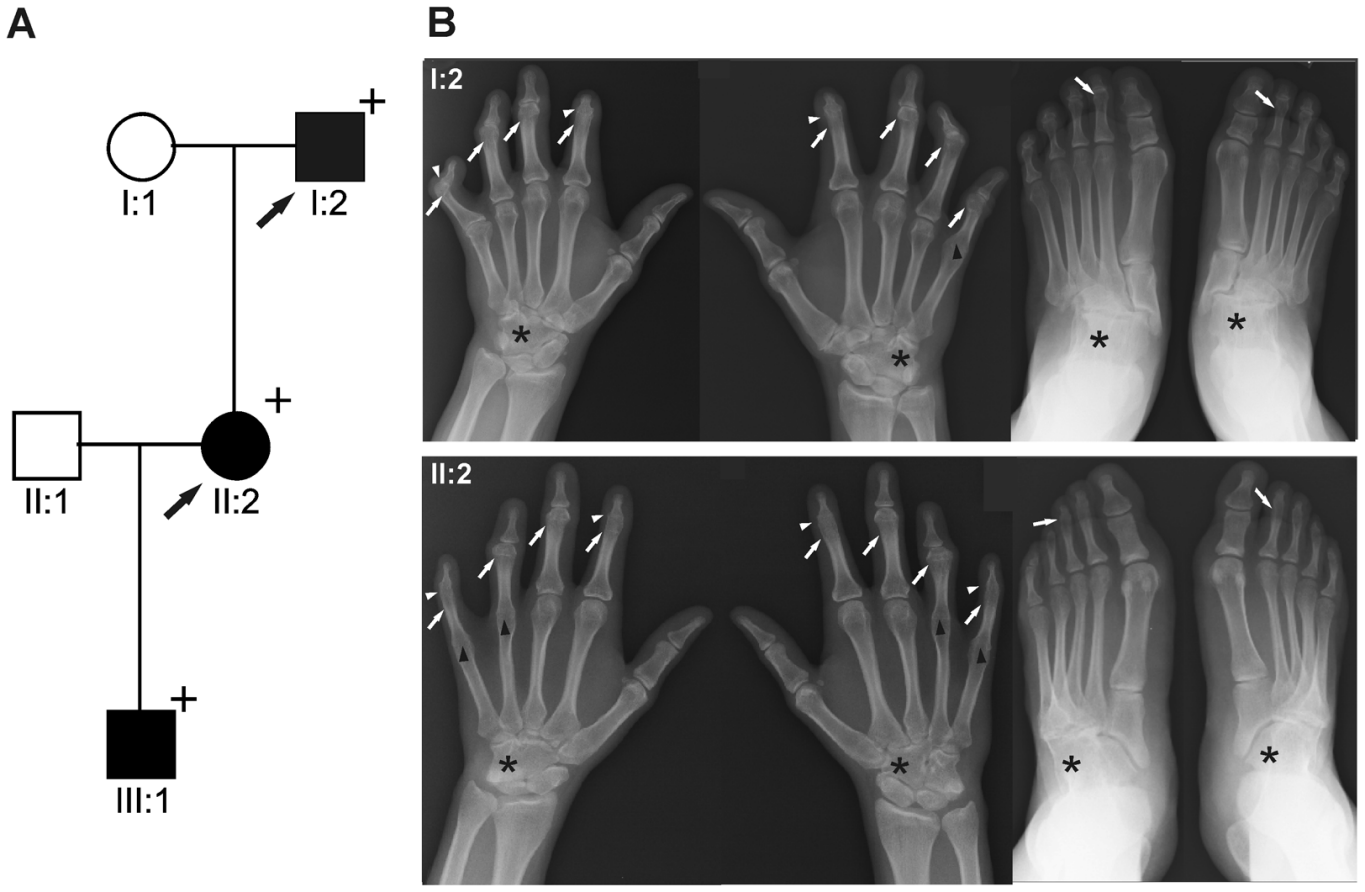
GDF5^W414R^ is associated with SYNS2 and BDA1. **A**: Pedigree of a family affected by SYNS2 and BDA1. Filled symbols represent affected family members and plus symbols indicate a confirmed mutation. Arrows identify the probands who underwent X-ray analysis. **B** Radiographs of hands and feet of individuals I:2 and II:2 displaying phenotypic abnormalities marked as follows: white arrows - proximal symphalangism of all fingers; arrowheads - distal symphalangism of the 2nd and 5th fingers; black arrowheads -synostoses of the 4th and 5th metacarpals with the corresponding proximal phalanges; asterisks - carpal and tarsal fusions. Overall, the fused or partially fused middle phalanges appear hypoplastic or rudimentary, consistent with BDA1. For a detailed list of phenotypic abnormalities observed in this family see also Table 1.

We report on a family of Mexican descent with an autosomal dominant form of SYNS2 with additional BDA1 (Figure 1A). Sequencing of the *GDF5* gene revealed a c.T1240C mutation (p.W414R) in three affected individuals from three generations. A mutation in *NOG*, a candidate gene for SYNS1, was excluded. The affected individuals are presented with multiple synostoses including proximal and distal symphalangism, metacarpophalangeal synostosis, and synostosis of carpal and tarsal bones as well as BDA1 with severe hypoplasia and even aplasia of the middle phalanges (Figure 1B and Table 1). Additional symptoms such as hearing impairment or short stature were not observed.

**Table 1:** Clinical features of the affected family members with mutations in *GDF5*.

Feature	HPO:ID	W414R	E491K [14]	R399C [19]
Proximal symphalangism	HP:0100264	**+**	**+**	−
Distal symphalangism	HP:0100263	**+**	**+**	−
Metacarpophalangeal Synostosis	HP:0100325	**+**	−	−
Synostosis of carpal bones	HP:0005048	**+**	**+**	−
Synostosis of tarsal bones	HP:0100330	**+**	**+**	−
Tarsometatarsal synostosis	HP:0100329	−	**+**	−
Aplasia/Hypoplasia of the middle phalanges of the hand (Brachydactyly Type A1)	HP:0009843	**+**	−	**+**
Hypoplastic/short 1^st^ metacarpal	HP:0010034	−	−	**+**

The features are coded using terms from the Human Phenotype Ontology [47]. + present; − absent. *GDF5* mutations are presented with either features of brachydactyly (GDF5 p.R399C) or features of synostosis (GDF5 p.E491K) or a combination of multiple synostosis with additional brachydactyly (GDF5 p.W414R).

### GDF5^W414R^ is positioned within the NOG and BMPRI binding interface

**Figure 2:**
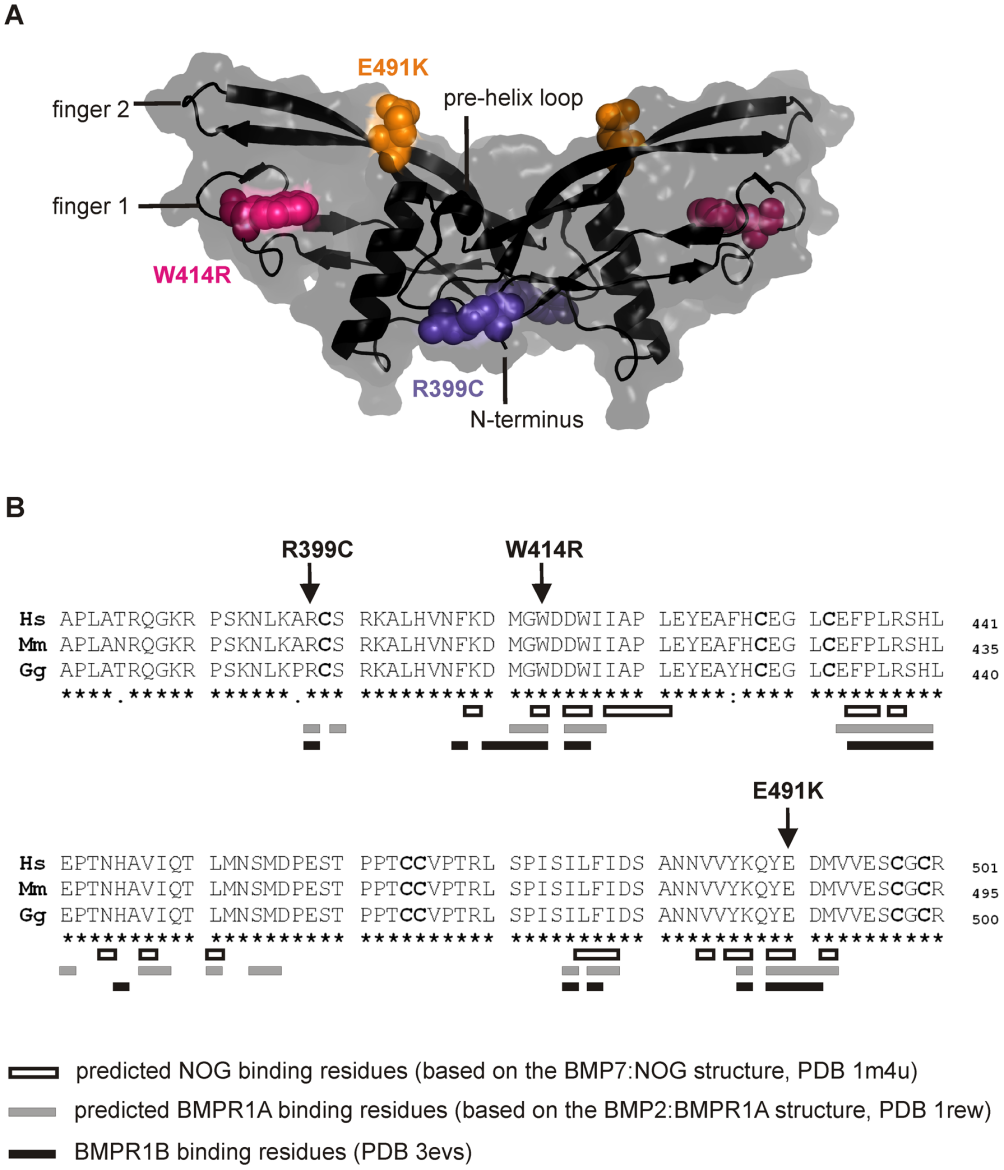
GDF5^W414R^ is positioned within the NOG and BMPR1A/B binding interface of the GDF5 dimer. **A**: 3D presentation of the human GDF5 homodimer (PDB 1waq). The topology of the GDF5 monomer comprises two ß-sheets forming the fingers as well as the four-turn α-helix with the preceding pre-helix loop. The mutations are highlighted in pink (GDF5^W414R^), violet (GDF5^R399C^) and orange (GDF5^E491K^). The image of the GDF5 structure was visualized using PyMol (http://www.pymol.org/). **B**: Protein sequence alignment of human, mouse and chicken GDF5 comprising the seven cysteine residues (bold) of the mature domain. Numbering is referred to the pro-protein sequence. Amino acids predicted to form the NOG binding interface are depicted as framed white boxes and based on the BMP7:NOG complex (PDB 1m4u). Residues predicted to be involved in BMPR1A binding are shown as grey boxes and refer to the BMP2:BMPR1A structure (PDB 1rew). Black boxes mark amino acids that bind to BMPR1B (PDB 3evs). Arrows indicate the mutated sites for GDF5^W414R^, GDF5^R399C^ and GDF5^E491K^. Note that GDF5^W414R^ and GDF5^E491K^ are located within the NOG binding site. Moreover, all three mutations interfere with the BMP type I receptor (BMPR1A and BMPR1B) binding interface.

The three mutations of interest (GDF5^W414R^, GDF5^R399C^, GDF5^E491K^) were highlighted in the GDF5 structure model (Figure 2A). GDF5^W414R^ is positioned within the long loop of finger 1, whereas GDF5^E491K^ is located within the second finger of the GDF5 dimer. GDF5^R399C^ is located at the N-terminal end, right in front of the β1 sheet of the first finger [23]. As shown in Figure 2B, all mutated sites in GDF5 are conserved among different species (human, mouse, chicken). Based on the crystal structures of the BMP7:NOG, BMP2:BMPR1A and GDF5:BMPR1B complexes, we predicted residues of GDF5 that are involved in binding to NOG or to the BMP type I receptors (Figure 2B) [5], [24]–[26]. Both SYNS2 associated variants, GDF5^W414R^ and GDF5^E491K^, are located within the NOG interaction site. Contrary, GDF5^R399C^, which is linked to an isolated BDA1 phenotype, is positioned outside of the NOG binding interface. Since all three mutations might also interfere with BMP type I receptor recruitment, we analyzed the interactions of the three mutations (GDF5^W414R^, GDF5^R399C^, GDF5^E491K^) to NOG and to BMPR1A and BMPR1B.

### GDF5^W414R^ is insensitive to inhibition by NOG

**Figure 3:**
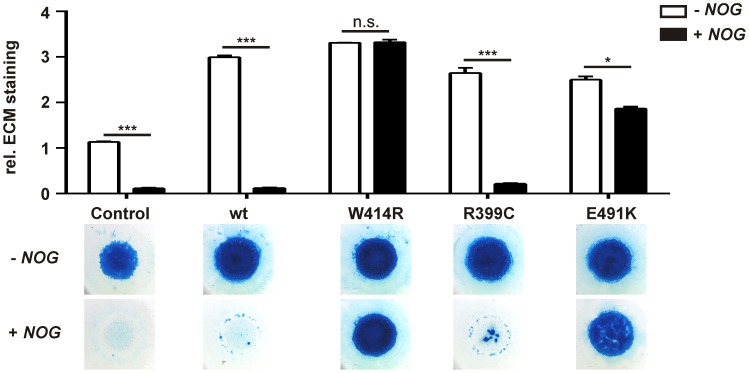
GDF5^W414R^ is resistant towards inhibition by NOG in chicken micromass cultures. Chicken micromass cells were infected with RCASBP(A) containing the coding sequence (cds) of either wild type *GDF5* or the GDF5 variants *GDF5^W414R^*, *GDF5^R399C^* or *GDF5^E491K^*. RCASBP(B) contained the cds of *NOG* and was used for co-transfection. Chicken micromass cultures and quantification of Alcian blue incorporation at 595 nm into the extracellular matrix (ECM) are shown for day 5. In the chicken micromass system, wild type GDF5 strongly induced chondrogenesis compared to the untransfected control. Chondrogenic differentiation was completely blocked in both, the control and wild type GDF5 cultures, when *NOG* is co-transfected. A similar pattern was observed for GDF5^R399C^. Contrary, GDF5^W414R^ and GDF5^E491K^ exhibited insensitivity towards the antagonist. Values represent the mean of triplicates and error bars indicate standard deviation. Statistical analysis was performed using a two-tailed Student's t test (n.s.: not significant; *p≤0.05; ***p≤0.001).

NOG, the main regulator of GDF5 activity, was initially identified to be mutated in patients with SYNS1 [27]. As GDF5^W414R^ is associated with the SYNS2 phenotype and furthermore located within the critical NOG binding site, we examined the signaling potency of the GDF5 mutations compared to wild type GDF5 in the absence and presence of NOG. We performed *in vitro* chondrogenesis assays and used the respective chicken *GDF5* constructs to infect chicken limb bud micromass cultures with and without *NOG*. Similar expression levels of wild type and mutant GDF5 were confirmed by Western blot (Figure S1, Text S1). As a chondrogenic marker, the extracellular matrix (ECM) produced by the limb bud cells was stained with Alcian blue (Figure 3).

In the absence of NOG, quantification of Alcian blue revealed a strong induction of early chondrogenesis for wild type GDF5 and GDF5^W414R^ as well as for the BDA1 causing variant GDF5^R399C^ and the SYM1/SYNS2-associated variant GDF5^E491K^. However, co-infection of *NOG* suppressed chondrogenesis effectively in wild type *GDF5* expressing cells, while *GDF5* ^W414R^ infected cells displayed a clear insensitivity towards NOG. NOG-resistance was also found for the GDF5^E491K^ variant. In contrast, cartilage formation was strongly inhibited in micromass cells expressing *GDF5* ^R399C^.

The reduced sensitivity of GDF5^W414R^ to NOG was also detected in Biacore measurements. In contrast to the high binding affinity of wild type GDF5 to NOG (apparent KD: ∼2 nM), GDF5^W414R^ showed a markedly reduced (∼12 fold) binding to NOG (apparent KD: ∼25.5 nM) (Table 2).

**Table 2:** Binding affinities of GDF5^W414R^ to immobilized receptor ectodomains.

KD [nM]	BMPR1A	BMPR1B	NOG
**GDF5^wt^**	17,0±4,6 [15]	1,1±0,2 [15]	2,0±0,6
**GDF^W414R^**	124,0±30,8	3,3±0,8	25,5±9,0

GDF5^W414R^ shows altered NOG and BMP receptor type I binding affinities in the Biacore assay.

Mean values from two experiments using at least six different analyte concentrations are shown.

### GDF5^W414R^ shows specific loss of BMPR1A signaling

**Figure 4:**
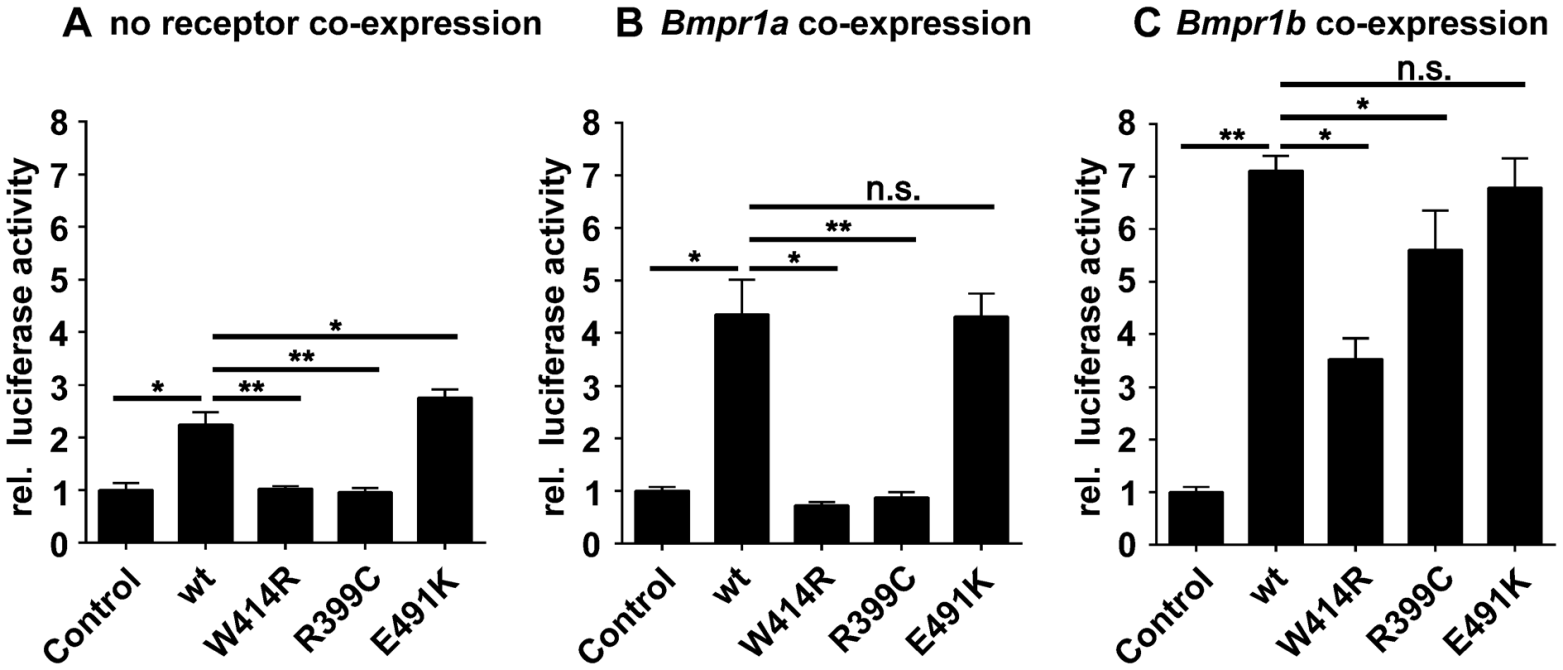
GDF5^W414R^ shows impaired Bmpr1a signaling in a SBE-Luciferase reporter gene assay. NIH/3T3 cells were transfected with the BMP type I receptors, *Bmpr1a* or *Bmpr1b,* as well as with wild type *GDF5* and the GDF5 variants *GDF5^W414R^*, *GDF5^R399C^* and *GDF5^E491K^*. As reporter, the SMAD binding element (SBE) was used and firely luciferase was normalized against TK-Renilla luciferase. **A**: No Bmp type I receptor was co-expressed which resulted in a weak SBE reporter activation for wild type GDF5 and GDF5^E491K^, whereas in case of GDF5^W414R^ and GDF5^R399C^ signaling activity was absent. **B**: *Bmpr1a* co-expression increased the signaling activity of wild type GDF5 and GDF5^E491K^; however, GDF5^W414R^ and GDF5^R399C^ were not able to induce reporter gene expression. **C**: Co-expression of *Bmpr1b* further increased the signaling activity of wild type GDF5 and GDF5^E491K^ compared to co-expression with *Bmpr1a*. In case of GDF5^W414R^ and GDF5^R399C^, *Bmpr1b* co-expression rescued their signaling activity. The means of triplicate measurements are shown, error bars indicate standard deviation and a represent experiment is shown. Statistical analysis was performed using a two-tailed Student's t test (n.s.: not significant; *p≤0.05; **p≤0.01). Significances are related to the respective wild type GDF5 value.

As GDF5^W414R^ is located in the overlapping interface of the high affinity BMP type I receptors and NOG, we analyzed subsequently signaling activities of wild type GDF5 and GDF5^W414R^ after co-expression of either one of the type I receptors, *Bmpr1a or Bmpr1b*. Signaling activities were determined in NIH/3T3 cells using a Smad Binding Element (SBE) luciferase reporter gene assay (Figure 4A–C).

Overexpression of wild type *GDF5* in combination with either one of the two type I receptors, *Bmpr1a* and *Bmpr1b*, resulted in a strong induction of luciferase activity. As expected from our Biacore data, wild type GDF5-induced signaling via Bmpr1b was stronger compared to signaling mediated via Bmpr1a (Figure 4B–C; Table 2). In case of GDF5^W414R^, no reporter gene activity was observed when *Bmpr1a* was additionally transfected (Figure 4B). However, co-transfection of *Bmpr1b* led to a clear induction of the SBE reporter, even though to a slightly lesser extent compared to wild type *GDF5* (Figure 4C). For the BDA1 associated variant GDF5^R399C^, we revealed the same signaling pattern in our luciferase assay as for GDF5^W414R^, which leads to the assumption that the pathomechanism of BDA1 is presumably connected with an alteration of the GDF5:BMPR1A binding interaction. In contrast, the SYM1/SYNS2 causing GDF5 variant GDF5^E491K^ promotes GDF5 signaling via Bmpr1a and Bmpr1b to a similar extent when compared to wild type GDF5.

Biacore analysis supported the findings from our cell based assays since GDF5^W414R^ showed a clear deviation from the wild type GDF5 receptor binding pattern. We could demonstrate a 7-fold lower affinity of GDF5^W414R^ to BMPR1A (apparent KD: ∼124 nM) compared to wild type GDF5 (apparent KD: ∼17 nM) and only a 3-fold lower affinity for BMPR1B (apparent KD: ∼3,3) compared to wild type GDF5 (apparent KD: ∼1,1 nM) (Table 2).

In summary, Biacore analysis and *in vitro* overexpression studies indicate a functional link between the phenotypic features of BDA1 and an impaired BMPR1A signaling of BDA1 associated GDF5 variants.

### Activity of GDF5^W414R^ is reduced in the absence of *Bmpr1b*

**Figure 5:**
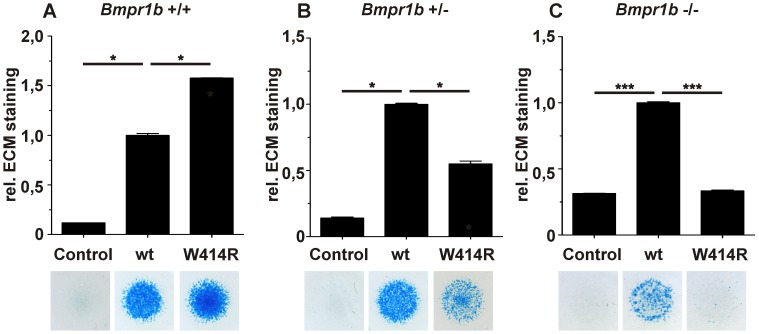
GDF5^W414R^ displays reduced chondrocyte differentiation in the absence of *Bmpr1b*. *Bmpr1b* wild type (*Bmpr1b* ^+/+^), heterozygous (*Bmpr1b* ^+/−^) and homozygous (*Bmpr1b* ^−/−^) mouse mesenchymal limb bud cells (E13.5) were stimulated with 5 nM recombinant human GDF5 protein (wild type GDF5 and GDF5^W414R^). Alcian blue incorporation into the extracellular matrix (ECM) was measured at 595 nm after five days of cultivation and four days of stimulation. **A**: Alcian blue staining of *Bmpr1b* ^+/+^ cells exhibited a strong induction of chondrogenesis upon stimulation with both recombinant GDF5 proteins. **B**: Stimulation of *Bmpr1b* ^+/−^ cells resulted in a reduced chondrogenic activity of GDF5^W414R^ compared to wild type GDF5. **C**: In case of *Bmpr1b* knockout cells, stimulation with GDF5^W414R^ resulted in a complete loss of chondrogenic activity, compared to wild type GDF5. Values represent the mean of three replicates, error bars indicate standard deviation. Statistical analysis was performed using a two-tailed Student's t test (*p≤0.05; ***p≤0.001).

In order to confirm the previous hypothesis, that GDF5^W414R^ is not able to transduce signaling via BMPR1A, we conducted an *in vitro* chondrogenesis assay using primary mesenchymal cells derived from *Bmpr1b* null mice (Figure 5A–C). Assuming that in wild type cells GDF5 signaling is mediated via Bmpr1a and Bmpr1b, a *Bmpr1b* knock-out would lead to a situation where solely Bmpr1a transmits GDF5-specific signals. Hence, we hypothesized that GDF5^W414R^ would not able to stimulate chondrogenic differentiation in cells lacking *Bmpr1b*, due to its insufficiency in binding to Bmpr1a.

As anticipated, heterozygous and homozygous *Bmpr1b* cells resulted in decreased chondrogenic activity for both, wild type GDF5 as well as GDF5^W414R^. However, wild type GDF5 stimulation led to an induction of chondrogenesis even in the complete absence of Bmpr1b, whereas GDF5^W414R^ stimulated cells displayed a complete loss of chondrogenic activity, indicating that a loss of binding of GDF5 to BMPR1A represents the centerpiece of the BDA1 pathomechanism.

### *Gdf5* expression co-localizes with *Nog* and *Bmpr1b* during limb development

To reconstruct the progress of GDF5 dependent limb development we analyzed the gene expression of *Gdf5* and its main antagonist *Nog* as well as its BMP type I receptors *Bmpr1a* and *Bmpr1b* in mice limb buds at stages E11.5 to E13.5, which represent critical phases of limb development.

**Figure 6:**
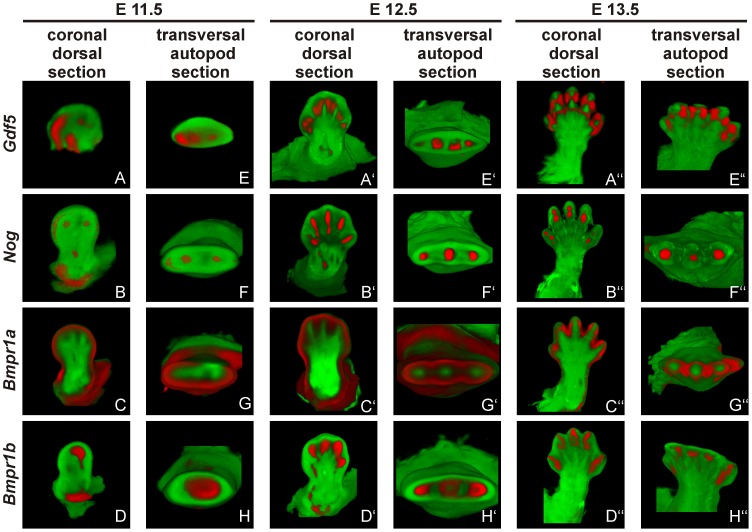
*Gdf5*, *Nog* and *Bmpr1b* are co-expressed during murine limb development. Mouse embryo*s* with the C57BL/6 genetic background at embryonic stages 11.5 (A–H), 12.5 (A′–H′) and 13.5 (A″–H″) were labeled with probes of *Gdf5* (A and E), *Nog* (B and F), *Bmpr1a* (C and G) or *Bmpr1b* (D and H) and signals are shown in red. Representatively, two sections of the coronal dorsal axis (A–D) and the autopod transversal axis (E–H) are depicted. The signal for *Gdf5* strongly co-localizes with the *Nog* and *Bmpr1b* expression pattern, whereas *Bmpr1a* expression is in direct proximity in the surrounding epithelium and underlying mesenchyme.

At stage E11.5, *Gdf5* is expressed in the anterior part of the limb bud (Figure 6A/E). Here, expression signals for *Nog* and *Bmpr1b* partly co-localize with *Gdf5* in the distal region (Figure 6B/F and D/H). Additionally, *Bmpr1b* and *Nog* show signals in the area of the later developing shoulder and *Nog* in the elbow joint as well. In contrast, *Bmpr1a* expression concentrates in the surrounding epithelium and underlying mesenchyme but sparing the central mesenchyme (Figure 6C/G). At stage E12.5 the expression pattern becomes more defined for *Gdf5*, *Nog* and *Bmpr1b* in the digital rays (Figure 6A′/E′, 6B′/F′ and 6D′/H′) and for *Bmpr1a* in direct proximity in the inter-phalangeal regions and the surrounding epithelium (Figure 6C′/G′). From stage E13.5 onwards, *Gdf5* expression concentrates in the joint interzones (Figure 6A″/E′), flanked by *Nog* and *Bmpr1b* expression (Figure 6B′/F′ and 6D′/H′). Apart from the distal tips, *Bmpr1a* is still expressed in the surrounding limb epithelium and interdigital mesenchyme.

The expression pattern analysis shows a co-localization for *Gdf5*, *Nog* and *Bmpr1b* and in case of *Bmpr1a*, expression in direct proximity to *Gdf5*.

## Discussion

Here we describe a novel GDF5 Trp to Arg transition (p.W414R) in patients with multiple synostoses syndrome 2 (SYNS2), including proximal and distal symphalangism, metacarpophalangeal synostosis, and synostosis of carpal and tarsal bones as well as BDA1 with severe hypoplasia and even aplasia of the middle phalanges. We identified that BDA1 and SYNS2 caused by GDF5^W414R^ are due to two independent molecular mechanisms involving specifically the BMP receptor BMPR1A and the BMP antagonist NOG, respectively.

**Figure 7:**
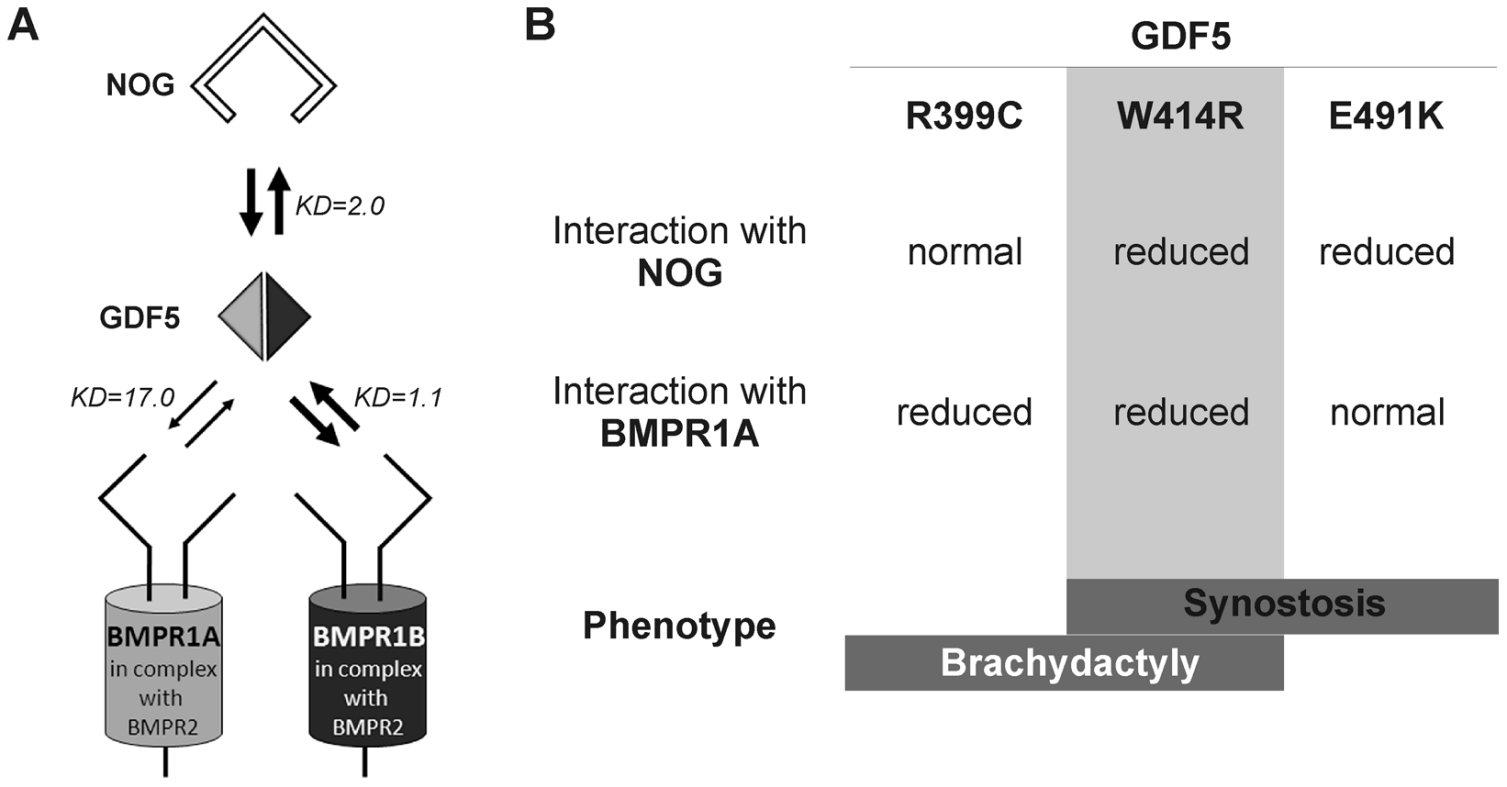
Disease model for SYNS2 and BDA1. **A**: During normal limb development, dimeric GDF5 (light/dark grey rhomb) is antagonized by NOG (black framed clamp) and thus balanced within the GDF5 signaling network. Downstream signaling is mediated via heteromeric receptor complexes consisting of each of the BMP type I receptors (BMPR1A and BMPR1B) in complex with the BMP type II receptor (BMPR2). Wild type GDF5 binds BMPR1A with a weaker affinity compared to BMPR1B as indicated by thin and thick arrows and additionally by Biacore binding affinities (KD). **B**: Summary of altered interaction of GDF5 mutations resulting in specific phenotypes. SYNS2 is characterized by GDF5 gain of function mutations, leading to an insensitivity of GDF5 towards its extracellular antagonist NOG. In contrast, BDA1 is caused by *GDF5* loss of function mutations, which result specifically in absent BMPR1A signaling.

Interestingly, mutations in *NOG* as well as in *GDF5* can lead to similar phenotypic characteristics of SYM1 and SYNS1/2 [15], [17], [27]. However, joint fusions caused by NOG mutations often affect the ossicles leading to hearing impairment, whereas mutations in GDF5 including GDF5^W414R^ spare this feature [27]–[29]. Regarding the literature, SYM1- and SYNS2-associated mutations in *GDF5* like GDF5^N445T^ and GDF5^S475N^ were shown to destabilize the GDF5/NOG interaction thus leading to a severe insensitivity towards the antagonist, also called NOG resistance [15], [17]. Therefore, we likewise analyzed GDF5^W414R^ concerning its interaction with NOG. W414 is located within the putative NOG binding interface, which was predicted based on the published superimposed GDF5:NOG complex [15], [30]. Interaction analyses of GDF5^W414R^ using chondrogenic differentiation assays together with Biacore binding studies revealed a NOG resistance as molecular cause of the joint fusion phenotype similar to GDF5^N445T^ and GDF5^S475N^ [15], [17]. In addition, we identified NOG insensitivity as the effect of the SYM1 associated GDF5^E491K^ mutation [14]. Hence, in case of SYM1 and SYNS2, an impaired GDF5/NOG interaction interferes with the negative feedback loop by which GDF5 is antagonized and thus balanced within the fine-tuned signaling network. Consequently, GDF5 variants associated with joint fusions exert an enhanced chondrogenic activity and can be referred to as gain of function mutations (Figure 7) [11]. The tight connection between GDF5 and NOG and their major importance for the development of joints become further visible as the results of our expression analyses of the developing mouse limb show overlapping temporal and spatial expression patterns of *Gdf5* and *Nog*.

To elucidate the underlying molecular mechanism by which GDF5^W414R^ causes joint fusions in combination with brachydactyly, we further analyzed how the mutation interferes with its cognate transmembrane BMP type I receptors. Situated in the long loop of finger 1 between the ß-sheets ß1/2 and ß3/ß4, W414 is positioned outside of the wrist epitope, which is mainly responsible for binding the BMP type I receptors BMPR1A and BMPR1B [5], [6], [23]. On the basis of the GDF5:BMPR1B crystal structure and the modeled GDF5:BMPR1A interaction, the contact of both BMP type I receptors with W414 was confirmed [5], [23]. As suggested, a transition of hydrophobic Trp to hydrophilic Arg at this highly conserved position results in impaired BMP type I receptor activation as shown in reporter gene assays and Biacore binding studies. Most strikingly, GDF5^W414R^ displayed a complete loss of BMPR1A activation, whereas signaling via BMPR1B was only moderately decreased. Possibly, a mutation interfering with the BMP type 1 receptor binding has in general a more drastic effect for the BMPR1A than for the BMPR1B, because the interaction with BMPR1A is per se lower. The remaining signaling activity of GDF5^W414R^ via BMPR1B seems to be sufficient to preserve its biological functionality as seen in our chondrogenic differentiation assays. A recently published GDF5 mutation (GDF5^R399C^) is likewise reported to cause BDA1. However, in this French Canadian family BDA1 occurs as an isolated trait in contrast to the phenotype of GDF5^W414R^, which is combined with features of synostoses [19]. GDF5^R399C^ is located at the N-terminus of the mature GDF5, right in front of the first β-sheet of finger 1, and is predicted to interfere with both BMP type I receptors [23]. Accordingly, we revealed a BMP type I receptor activation pattern similar to that of GDF5^W414R^ indicating that the disruption of BMPR1A signaling is a hallmark of the BDA1 pathomechanism (Figure 7).

There are two studies which deal explicit with the analyses of specific functions of BMPR1A and BMPR1B, one was done in chicken and the other one was done in mice [31], [32]. In the chicken study the expression patterns of both receptors were distinct during limb development. BMPR1B was strongly expressed in precartilaginous condensations, whereas BMPR1A was reported to be expressed throughout the limb mesenchyme. To rule out a functional difference between BMPR1A and BMPR1B, the authors overexpressed either constitutive active (c.a.) or dominant negative (d.n.) variants of BMPR1A and BMPR1B *in vivo* in the chicken limb bud or *in vitro* in chicken micromass cultures [32]. Interestingly, BMPR1B turned out to be necessary for early steps of cartilage formation, whereas BMPR1A was shown to elicit an important function in prehypertrophic chondrocytes. Expression of c.a. BMPR1A led to a delay of chondrogenic differentiation; similar to the phenotype caused by overexpression of IHH [33]. Another study indicated that the IHH-regulated process of chondrogenic differentiation indeed requires BMP signaling [34]. As both signals, IHH and BMP, are required for maintaining a normal proliferation rate and regular differentiation of chondrocytes, these findings could explain how BDA1 can be caused by mutations in IHH or the BMP pathway. A few years later a study was undertaken in mice, where authors concluded that Bmpr1a and Bmpr1b have mostly redundant functions in chondrogenesis [31]. This statement was made due to the observation that single knock outs of either Bmpr1a or Bmpr1b showed only subtle skeletal phenotypes, whereas the double knock out displayed a very strong phenotype with a nearly absent endochondral skeleton. Nevertheless, the phenotypes of each knock out are very distinct, for example Bmpr1b null mice displayed defects in the appendicular skeleton, whereas Bmpr1a cKO shows a generalized chondrodysplasia and the more severe phenotype seen in the double knock out could also be explained by an additive or synergistic effect. Therefore we suggest that both receptors have unique functions and loss of binding of the GDF5 mutants to one of the two receptors cannot be compensated by the other receptor.

Loss of GDF5 receptor binding in general plays a central role within the molecular disease family of brachydactylies. For example, the GDF5 variant GDF5^L441P^ causes BDA2 due to an impaired BMPR1B binding [11], [16]. Vice versa, specific mutations in *BMPR1B* are associated with BDA2 [35]. Compared to BDA1, where all middle phalanges are affected and distal symphalangism can occur, BDA2 is characterized by short or absent middle phalanges only of the second and sometimes fifth finger. BDC comprises features of BDA1 and BDA2 and primarily affects the middle phalanges of the second, third and fifth fingers and the first metacarpal bone. Interestingly, the molecular reason for BDC is functional haploinsufficiency of GDF5 [21]. The implication of GDF5 in chondrogenesis and joint formation can finally be highlighted in connection with osteoarthritis (OA; MIM #165720), the most common form of late-onset destruction of articular cartilage in synovial joints nowadays [36]. Among various genetic loci, GDF5 has been discovered as the most consistent and robust risk factor of OA, whereby decreased Gdf5 mRNA levels have been found to account for a murine OA-like phenotype [36]–[38]. Furthermore Masuya et al. identified a Trp to Arg transition in a large ENU mutagenesis screen, which was described to impair joint formation and thereby cause OA [39]. This mutation (p.W408R) is the mouse homologue to the GDF5^W414R^ mutation we described in this work. Therefore, understanding the biology of GDF5^W414R^ might also give insights into the pathophysiology of OA.

In summary, we revealed that GDF5^W414R^, in contrast to wild type GDF5, loses the BMPR1A signaling route and at the same time increases the alternative signaling via BMPR1B in the presence of NOG. Therefore, the reduced sensitivity of W414R to Noggin and its reduced interaction with BMPR1A do not actually “neutralize” each other, but lead to a misbalance of BMPR1A and BMPR1B signaling. Hence, our study assembles another part of the molecular puzzle how loss and gain of function mutations in GDF5 affect bone development in hands and feet and result in specific types of brachydactyly and SYNS2.

## Materials and Methods

### Clinical investigation and molecular analysis

All clinical investigations have been performed according to Declaration of Helsinki principles. The study was approved by the local institutional review board “Ethikkommission der Charité - Universitätsmedizin Berlin”. Informed consent for genetic testing was obtained from the patient or their legal guardians respectively. Genomic DNA of affected family members were extracted from peripheral blood samples by standard methods. The coding regions of *NOG* and *GDF5* as well as the flanking intronic sequences were amplified by standard PCR protocols. The primer sequences and PCR conditions for the molecular testing were previously described [20], [30]. PCR products were analyzed on 2% agarose gels. Sequencing was done using the ABI Prism BigDye Terminator Sequencing Kit (Applied Biosystems) with PCR primers used as sequencing primers. Products were evaluated on an automated capillary sequencer (Applied Biosystems).

### Chicken micromass cultures

Cloning of the coding sequences of chicken *GDF5* and *NOG* into RCAS(BP)A or RCAS(BP)B, respectively was previously described [15]. Mutations (GDF5^W414R^, GDF5^R399C^, GDF5^E491K^) were introduced into the coding sequence of chicken *GDF5* in pSLAX13 by *in vitro* mutagenesis. Primer sequences are available in the supplement (Table S1). Production of viral supernatant in DF1 cells and concentration of viral particles was performed as described previously [40]. Fertilized chicken eggs were obtained from VALO BioMedia GmbH (Osterholz-Scharmbeck, Germany) and incubated at 38°C in a humidified egg incubator for 4.5 days. Micromass cultures were plated in a drop containing 2×10^5^ cells. Infection was performed with concentrated viral supernatants: RCASBP(A) containing cDNA encoding chicken wild type *GDF5* and the *GDF5* mutants *GDF5^W414R^*, *GDF5^R399C^*, and *GDF5^E491K^* with a titer of 1×10^7^ plaque forming units (PFU)/ml. RCASBP(B) containing the cDNA encoding chicken wild type *NOG* was applied with a titer of 2,5×10^6^ PFU/ml. Culture medium containing DMEM-F12 (Biochrom), 10% FBS (Biochrom), 0,2% chicken serum (Sigma), 2 mM L-Gln (Lonza), 100 U/ml penicillin, and 100 µg/ml streptomycin (Lonza) was replaced every 2 days. For each condition, three replicates were performed in parallel. Quantification of Alcian blue dye was performed at 595 nm after extraction with Guanidin-HCl.

### Recombinant proteins

Recombinant human (rh) GDF5 and its variant rhGDF5^W414R^ were dissolved in 10 mM HCl and provided by Biopharm GmbH.

### BIAcore binding assay

The BIA2000 system (Biacore) was used to analyze the binding affinities of recombinant human GDF5 and its variant GDF5^W414R^ to immobilized NOG and ectodomains of BMPR1A, BMPR1B and BMPR2, as previously described [16].

### Luciferase activity assay

Coding sequences of human *GDF5* and mouse *Bmpr1a* and *Bmpr1b* were cloned into pSLAX13. Mutations (GDF5^W414R^, GDF5^R399C^, GDF5^E491K^) were introduced into the coding sequence of human *GDF5* in pSLAX13 by *in vitro* mutagenesis. Primer sequences are available in the supplement (Table S1). Inserts were subcloned into pCS2+ via ClaI.

Luciferase reporter gene assays were performed using the murine fibroblast cell line NIH/3T3 (ATCC) which was maintained in DMEM high glucose (Lonza) with 10% FCS (Biochrom), 2 mM L-Gln (Lonza), 100 U/ml penicillin, and 100 µg/ml streptomycin (Lonza). Prior to transfection, cells were seeded in a 96-well plate at a density of 1×10^4^ cells per well. BMP receptors and GDF5 constructs were transfected for 40 hours together with the Smad Binding Element luciferase construct SBE-pGL3 [41] and the normalization vector pRLTk (Promega) using Lipofectamine 2000 (Invitrogen). Luciferase activity was determined as described previously [42].

### Mouse micromass cultures

Limb mesenchymal cells were isolated from stage E13.5 embryos resulting from matings of C57BL/6, Bmpr1b^tm1kml^ heterozygous or homozygous knock-out mice on a C57BL/6 background [43]. Mouse embryos were genotyped using primers for *Bmpr1b* and *neomycin* (Table S2), if applicable embryos were pooled according to their phenotypes. Isolation of mouse micromass cells was performed as described for chicken micromass cultures with minor modifications. For mouse micromass cultures no additional chicken serum was used. After 24 h mouse micromass cultures were stimulated with 5 nM of recombinant human wild type GDF5 and GDF5^W414R^.

### Whole mount *in situ* hybridization

C57BL/6 mouse embryos were harvested at stages E11.5–13.5 and fixed in 4% PFA. Whole mount *in situ* hybridization was performed as previously described [44].

DIG-labeled RNA antisense-probes were generated by *in vitro* transcription using the coding sequences of mouse *Bmpr1a*, *Bmpr1b* and *Nog* as a template. The probe for mouse *Gdf5* was previously published [45]. Signal detection was performed with BMPurple (Roche). 3D imaging of labeled limbs was done by optical projection tomography (OPT) scans as previously described [46].

### Statistics

Statistical analyses were performed using a two-tailed Student's T-test. Results are presented as mean ± SEM. P values of less than 0.05 were considered significant.

## Supporting Information

Figure S1 Wild type and mutant GDF5 transcripts are expressed at comparable levels in chicken micromass cultures. Chicken micromass cultures were infected with empty RCASBP(A) as control and RCASBP(A) containing the cds of either wild type *GDF5* or the GDF5 variants (*GDF5^W414R^*, *GDF5^R399C^*, *GDF5^E491K^*). After SDS-PAGE under non-reducing (GDF5) and reducing (ACTIN) conditions and subsequent Western Blot, GDF5 and ACTIN were detected at comparable levels using specific antibodies.
(TIF)

Table S1 Primers used for site-directed mutagenesis. *In vitro* mutagenesis of GDF5 mutations (GDF5^W414R^, GDF5^R399C^, GDF5^E491K^) into the coding sequences of chicken *GDF5* and human *GDF5* were carried out by using the following primers.
(DOC)

Table S2 Primers used for mouse genotyping. Genotyping of *Bmpr1b* wild type (*Bmpr1b* ^+/+^), heterozygous (*Bmpr1b* ^+/−^) and homozygous (*Bmpr1b* ^−/−^) mouse embryos for mouse micromass assays was carried out using the following primers.
(DOC)

Text S1 Materials and Methods for anti-GDF5 Western blot.
(DOC)

## References

[1] Chang SC, Hoang B, Thomas JT, Vukicevic S, Luyten FP, et al (1994) Cartilage-derived morphogenetic proteins. New members of the transforming growth factor-beta superfamily predominantly expressed in long bones during human embryonic development. J Biol Chem 269: 28227–28234. 7961761

[2] Stricker S, Mundlos S (2011) Mechanisms of Digit Formation: Human Malformation Syndromes Tell the Story. Developmental Dynamics 240: 990–1004. 21337664 10.1002/dvdy.22565

[3] Buxton P, Edwards C, Archer CW, Francis-West P (2001) Growth/differentiation factor-5 (GDF-5) and skeletal development. J Bone Joint Surg Am 83-A Suppl 1: S23–30. 11263662

[4] Storm EE, Huynh TV, Copeland NG, Jenkins NA, Kingsley DM, et al (1994) Limb alterations in brachypodism mice due to mutations in a new member of the TGF beta-superfamily. Nature 368: 639–643. 8145850 10.1038/368639a0

[5] Kotzsch A, Nickel J, Seher A, Sebald W, Muller TD (2009) Crystal structure analysis reveals a spring-loaded latch as molecular mechanism for GDF-5-type I receptor specificity. EMBO J 28: 937–947. 19229295 10.1038/emboj.2009.37 PMC2670865

[6] Mueller TD, Nickel J (2012) Promiscuity and specificity in BMP receptor activation. FEBS Lett 586: 1846–1859. 22710174 10.1016/j.febslet.2012.02.043

[7] Nohe A, Keating E, Knaus P, Petersen NO (2004) Signal transduction of bone morphogenetic protein receptors. Cell Signal 16: 291–299. 14687659 10.1016/j.cellsig.2003.08.011

[8] Schmierer B, Hill CS (2007) TGFbeta-SMAD signal transduction: molecular specificity and functional flexibility. Nat Rev Mol Cell Biol 8: 970–982. 18000526 10.1038/nrm2297

[9] Miyazono K, Kamiya Y, Morikawa M (2010) Bone morphogenetic protein receptors and signal transduction. J Biochem 147: 35–51. 19762341 10.1093/jb/mvp148

[10] Bragdon B, Moseychuk O, Saldanha S, King D, Julian J, et al (2011) Bone morphogenetic proteins: a critical review. Cell Signal 23: 609–620. 20959140 10.1016/j.cellsig.2010.10.003

[11] Mundlos S (2009) The brachydactylies: a molecular disease family. Clin Genet 76: 123–136. 19790289 10.1111/j.1399-0004.2009.01238.x

[12] Lories RJ, Luyten FP (2005) Bone morphogenetic protein signaling in joint homeostasis and disease. Cytokine Growth Factor Rev 16: 287–298. 15993360 10.1016/j.cytogfr.2005.02.009

[13] Dawson K, Seeman P, Sebald E, King L, Edwards M, et al (2006) GDF5 is a second locus for multiple-synostosis syndrome. Am J Hum Genet 78: 708–712. 16532400 10.1086/503204 PMC1424701

[14] Wang X, Xiao F, Yang Q, Liang B, Tang Z, et al (2006) A novel mutation in GDF5 causes autosomal dominant symphalangism in two Chinese families. Am J Med Genet A 140A: 1846–1853. 16892395 10.1002/ajmg.a.31372

[15] Seemann P, Brehm A, Konig J, Reissner C, Stricker S, et al (2009) Mutations in GDF5 reveal a key residue mediating BMP inhibition by NOGGIN. PLoS Genet 5: e1000747. 19956691 10.1371/journal.pgen.1000747 PMC2776984

[16] Seemann P, Schwappacher R, Kjaer KW, Krakow D, Lehmann K, et al (2005) Activating and deactivating mutations in the receptor interaction site of GDF5 cause symphalangism or brachydactyly type A2. J Clin Invest 115: 2373–2381. 16127465 10.1172/JCI25118 PMC1190374

[17] Schwaerzer GK, Hiepen C, Schrewe H, Nickel J, Ploeger F, et al (2012) New insights into the molecular mechanism of multiple synostoses syndrome (SYNS): mutation within the GDF5 knuckle epitope causes noggin-resistance. J Bone Miner Res 27: 429–442. 21976273 10.1002/jbmr.532

[18] Ploger F, Seemann P, Schmidt-von Kegler M, Lehmann K, Seidel J, et al (2008) Brachydactyly type A2 associated with a defect in proGDF5 processing. Hum Mol Genet 17: 1222–1233. 18203755 10.1093/hmg/ddn012

[19] Byrnes AM, Racacho L, Nikkel SM, Xiao F, MacDonald H, et al (2010) Mutations in GDF5 presenting as semidominant brachydactyly A1. Hum Mutat 31: 1155–1162. 20683927 10.1002/humu.21338

[20] Schwabe GC, Turkmen S, Leschik G, Palanduz S, Stover B, et al (2004) Brachydactyly type C caused by a homozygous missense mutation in the prodomain of CDMP1. Am J Med Genet A 124A: 356–363. 14735582 10.1002/ajmg.a.20349

[21] Everman DB, Bartels CF, Yang Y, Yanamandra N, Goodman FR, et al (2002) The mutational spectrum of brachydactyly type C. Am J Med Genet 112: 291–296. 12357473 10.1002/ajmg.10777

[22] Thomas JT, Kilpatrick MW, Lin K, Erlacher L, Lembessis P, et al (1997) Disruption of human limb morphogenesis by a dominant negative mutation in CDMP1. Nat Genet 17: 58–64. 9288098 10.1038/ng0997-58

[23] Nickel J, Kotzsch A, Sebald W, Mueller TD (2005) A single residue of GDF-5 defines binding specificity to BMP receptor IB. J Mol Biol 349: 933–947. 15890363 10.1016/j.jmb.2005.04.015

[24] Keller S, Nickel J, Zhang JL, Sebald W, Mueller TD (2004) Molecular recognition of BMP-2 and BMP receptor IA. Nat Struct Mol Biol 11: 481–488. 15064755 10.1038/nsmb756

[25] Kirsch T, Sebald W, Dreyer MK (2000) Crystal structure of the BMP-2-BRIA ectodomain complex. Nat Struct Biol 7: 492–496. 10881198 10.1038/75903

[26] Groppe J, Greenwald J, Wiater E, Rodriguez-Leon J, Economides AN, et al (2002) Structural basis of BMP signalling inhibition by the cystine knot protein Noggin. Nature 420: 636–642. 12478285 10.1038/nature01245

[27] Gong Y, Krakow D, Marcelino J, Wilkin D, Chitayat D, et al (1999) Heterozygous mutations in the gene encoding noggin affect human joint morphogenesis. Nat Genet 21: 302–304. 10080184 10.1038/6821

[28] Brown DJ, Kim TB, Petty EM, Downs CA, Martin DM, et al (2002) Autosomal dominant stapes ankylosis with broad thumbs and toes, hyperopia, and skeletal anomalies is caused by heterozygous nonsense and frameshift mutations in NOG, the gene encoding noggin. Am J Hum Genet 71: 618–624. 12089654 10.1086/342067 PMC379196

[29] Mangino M, Flex E, Digilio MC, Giannotti A, Dallapiccola B (2002) Identification of a novel NOG gene mutation (P35S) in an Italian family with symphalangism. Hum Mutat 19: 308. 11857750 10.1002/humu.9016

[30] Lehmann K, Seemann P, Silan F, Goecke TO, Irgang S, et al (2007) A new subtype of brachydactyly type B caused by point mutations in the bone morphogenetic protein antagonist NOGGIN. Am J Hum Genet 81: 388–396. 17668388 10.1086/519697 PMC1950796

[31] Yoon BS, Ovchinnikov DA, Yoshii I, Mishina Y, Behringer RR, et al (2005) Bmpr1a and Bmpr1b have overlapping functions and are essential for chondrogenesis in vivo. Proc Natl Acad Sci U S A 102: 5062–5067. 15781876 10.1073/pnas.0500031102 PMC555995

[32] Zou H, Wieser R, Massague J, Niswander L (1997) Distinct roles of type I bone morphogenetic protein receptors in the formation and differentiation of cartilage. Genes Dev 11: 2191–2203. 9303535 10.1101/gad.11.17.2191 PMC275391

[33] Vortkamp A, Lee K, Lanske B, Segre GV, Kronenberg HM, et al (1996) Regulation of rate of cartilage differentiation by Indian hedgehog and PTH-related protein. Science 273: 613–622. 8662546 10.1126/science.273.5275.613

[34] Minina E, Wenzel HM, Kreschel C, Karp S, Gaffield W, et al (2001) BMP and Ihh/PTHrP signaling interact to coordinate chondrocyte proliferation and differentiation. Development 128: 4523–4534. 11714677 10.1242/dev.128.22.4523

[35] Lehmann K, Seemann P, Stricker S, Sammar M, Meyer B, et al (2003) Mutations in bone morphogenetic protein receptor 1B cause brachydactyly type A2. Proc Natl Acad Sci U S A 100: 12277–12282. 14523231 10.1073/pnas.2133476100 PMC218749

[36] Reynard LN, Loughlin J (2012) Genetics and epigenetics of osteoarthritis. Maturitas 71: 200–204. 22209350 10.1016/j.maturitas.2011.12.001

[37] Liu J, Cai W, Zhang H, He C, Deng L (2013) Rs143383 in the Growth Differentiation Factor 5 (GDF5) Gene Significantly Associated with Osteoarthritis (OA)-A Comprehensive Meta-analysis. Int J Med Sci 10: 312–319. 23423687 10.7150/ijms.5455 PMC3575627

[38] Daans M, Luyten FP, Lories RJ (2011) GDF5 deficiency in mice is associated with instability-driven joint damage, gait and subchondral bone changes. Ann Rheum Dis 70: 208–213. 20805298 10.1136/ard.2010.134619

[39] Masuya H, Nishida K, Furuichi T, Toki H, Nishimura G, et al (2007) A novel dominant-negative mutation in Gdf5 generated by ENU mutagenesis impairs joint formation and causes osteoarthritis in mice. Hum Mol Genet 16: 2366–2375. 17656374 10.1093/hmg/ddm195

[40] Morgan BA, Fekete DM (1996) Manipulating gene expression with replication-competent retroviruses. Methods Cell Biol 51: 185–218. 8722477 10.1016/s0091-679x(08)60629-9

[41] Jonk LJ, Itoh S, Heldin CH, ten Dijke P, Kruijer W (1998) Identification and functional characterization of a Smad binding element (SBE) in the JunB promoter that acts as a transforming growth factor-beta, activin, and bone morphogenetic protein-inducible enhancer. J Biol Chem 273: 21145–21152. 9694870 10.1074/jbc.273.33.21145

[42] Hampf M, Gossen M (2006) A protocol for combined Photinus and Renilla luciferase quantification compatible with protein assays. Anal Biochem 356: 94–99. 16750160 10.1016/j.ab.2006.04.046

[43] Yi SE, Daluiski A, Pederson R, Rosen V, Lyons KM (2000) The type I BMP receptor BMPRIB is required for chondrogenesis in the mouse limb. Development 127: 621–630. 10631182 10.1242/dev.127.3.621

[44] Pryce BA, Brent AE, Murchison ND, Tabin CJ, Schweitzer R (2007) Generation of transgenic tendon reporters, ScxGFP and ScxAP, using regulatory elements of the scleraxis gene. Dev Dyn 236: 1677–1682. 17497702 10.1002/dvdy.21179

[45] Sharpe J, Ahlgren U, Perry P, Hill B, Ross A, et al (2002) Optical projection tomography as a tool for 3D microscopy and gene expression studies. Science 296: 541–545. 11964482 10.1126/science.1068206

[46] Quintana L, Sharpe J (2011) Preparation of mouse embryos for optical projection tomography imaging. Cold Spring Harb Protoc 2011: 664–669. 21632773 10.1101/pdb.prot5639

[47] Robinson PN, Kohler S, Bauer S, Seelow D, Horn D, et al (2008) The Human Phenotype Ontology: a tool for annotating and analyzing human hereditary disease. Am J Hum Genet 83: 610–615. 18950739 10.1016/j.ajhg.2008.09.017 PMC2668030

