# Supplementary figures and images for: A GDF5 Point Mutation Strikes Twice - Causing BDA1 and SYNS2
Source: PLoS Genet. 2013 Oct 3;9(10):e1003846. doi: 10.1371/journal.pgen.1003846 (PMC3789827; doi:10.1371/journal.pgen.1003846)

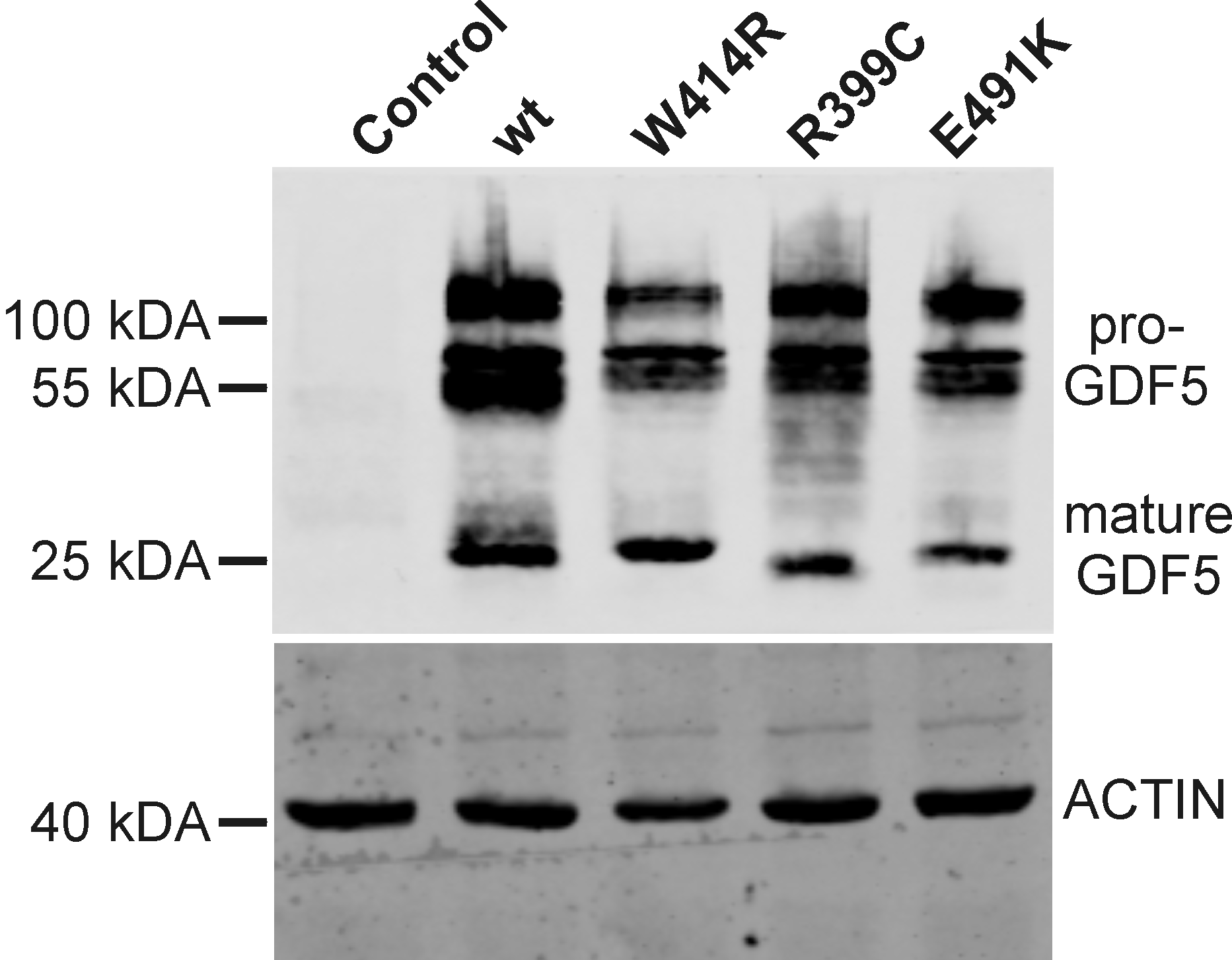

Supplement: Figure S1 — Wild type and mutant GDF5 transcripts are expressed at comparable levels in chicken micromass cultures. Chicken micromass cultures were infected with empty RCASBP(A) as control and RCASBP(A) containing the cds of either wild type GDF5 or the GDF5 variants (GDF5W414R, GDF5R399C, GDF5E491K). After SDS-PAGE under non-reducing (GDF5) and reducing (ACTIN) conditions and subsequent Western Blot, GDF5 and ACTIN were detected at comparable levels using specific antibodies. (TIF) [file pgen.1003846.s001.tif]
