# Supplementary material for: A GDF5 Point Mutation Strikes Twice - Causing BDA1 and SYNS2
Source: PLoS Genet. 2013 Oct 3;9(10):e1003846. doi: 10.1371/journal.pgen.1003846 (PMC3789827; doi:10.1371/journal.pgen.1003846)
Supplement: Table S1 — Primers used for site-directed mutagenesis. In vitro mutagenesis of GDF5 mutations (GDF5W414R, GDF5R399C, GDF5E491K) into the coding sequences of chicken GDF5 and human GDF5 were carried out by using the following primers. (DOC) [file pgen.1003846.s002.doc]

**Table S1**

| **Primer name** | **Primer sequence** |
| --- | --- |
| GDF5_W414R_fwd (human) | CAACTTCAAGGACATGGGCcGGGACGACTGGATCATCGC |
| GDF5_W414R_rev (human) | GCGATGATCCAGTCGTCCCgGCCCATGTCCTTGAAGTTG |
| Gdf5_W414R_fwd (chicken) | gaattttaaggacatgggccgggatgactggataatagc |
| Gdf5_W414R_rev (chicken) | gctattatccagtcatcccggcccatgtccttaaaattc |
| GDF5_R399C_fwd (human) | GCAAGAACCTTAAGGCTtGCTGCAGTCGGAAGGCAC |
| GDF5_R399C_rev (human) | GTGCCTTCCGACTGCAGCaAGCCTTAAGGTTCTTGC |
| Gdf5_R399C_fwd (chicken) | CCAGTAAGAACCTGAAGGCAtGcTGTAGCAGAAAAGCCC |
| Gdf5_R399C_rev (chicken) | GGGCTTTTCTGCTACAgCaTGCCTTCAGGTTCTTACTGG |
| GDF5_E491K_fwd (human) | GCCCACGCGGCTGAGTCCCATCAaCATCCTCTTCATTGACTCTGC |
| GDF5_E491K_rev (human) | GCAGAGTCAATGAAGAGGATGtTGATGGGACTCAGCCGCGTGGGC |
| Gdf5_E491K_fwd (chicken) | tggtctacaagcagtacaaggacatggtggtggagtcgtg |
| Gdf5_E491K_rev (chicken) | cacgactccaccaccatgtccttgtactgcttgtagacca |
