# Supplementary material for: A GDF5 Point Mutation Strikes Twice - Causing BDA1 and SYNS2
Source: PLoS Genet. 2013 Oct 3;9(10):e1003846. doi: 10.1371/journal.pgen.1003846 (PMC3789827; doi:10.1371/journal.pgen.1003846)
Supplement: Table S2 — Primers used for mouse genotyping. Genotyping of Bmpr1b wild type (Bmpr1b +/+), heterozygous (Bmpr1b +/−) and homozygous (Bmpr1b −/−) mouse embryos for mouse micromass assays was carried out using the following primers. (DOC) [file pgen.1003846.s003.doc]

**Table S2**

| **Primer name** | **Primer sequence** |
| --- | --- |
| Bmpr1b_Flox_fwd (mouse) | TGGTGAGTGGTTACAACAAGATCAGCA |
| Bmpr1b_Flox_rev (mouse) | CTCGGCCCAAGATCCTACGTTG |
| Bmpr1b_Neo_fwd (mouse) | TTGTCACTGAAGCGGGAAGG |
| Bmpr1b_Neo_rev (mouse) | TTGAGCCTGGCGAACAGTTC |
