# Supplementary material for: A GDF5 Point Mutation Strikes Twice - Causing BDA1 and SYNS2
Source: PLoS Genet. 2013 Oct 3;9(10):e1003846. doi: 10.1371/journal.pgen.1003846 (PMC3789827; doi:10.1371/journal.pgen.1003846)
Supplement: Text S1 — Materials and Methods for anti-GDF5 Western blot. (DOC) [file pgen.1003846.s004.doc]

**Text S1**

Western Blot analysis was performed as previously described with minor changes . In brief, at day 5 chicken micromass cultures were lysed in lysis buffer (50 mM HEPES, 50 mM NaCl, 10 mM EDTA, 10% glycerol, 1% Triton, 100 mM PMSF) and homogenized using a TissueLyser (Qiagen). Samples were separated under non-reducing (GDF5) and reducing (Actin) conditions using 10% SDS-PAGE. Immunodetection was performed using a mouse anti-GDF5 antibody (Biopharm) and a rabbit anti-Actin antibody (A2066, Sigma-Aldrich) as primary antibodies. Signals were detected via IRDye labeled secondary antibodies (IRDye goat anti mouse 800; IRDye goat anti rabbit 680, LICOR).

1. Ploger F, Seemann P, Schmidt-von Kegler M, Lehmann K, Seidel J, Kjaer KW, Pohl J, Mundlos S 2008 Brachydactyly type A2 associated with a defect in proGDF5 processing. Hum Mol Genet **17**(9)**:**1222-1233.
